# The Relative Value of Technical Instruction

**Published:** 1898-04

**Authors:** 


### Editorial.

### THE RELATIVE VALUE OF TECHNICAL INSTRUCTION.

WHEN the proceedings of four years of the National School of Dental Technics fell into our hands, it was with the hope that something valuable would be gained through the experience of many teachers in this work, in which all dental educators have a direct interest. In some respects this hope has been fulfilled, but, it must be confessed, not to the extent anticipated.

The report presents to the mind a series of papers, many valuable in suggestive thought and practice, and all tending to make college work of more value than it was in the earlier period of dental education. This effort is worthy of all praise, as all attempts at an honest advance in any calling must ever be, and for this due credit should be given.

The establishment of this association of dental pedagogics—for such it is—began in the needs of the more isolated colleges in the Middle West, and has found its most ardent supporters in that region. The reason for this is not difficult to determine, it having been the result of an increasing difficulty in keeping the growing numbers of students fully occupied during the scholastic term.

Many of the schools had been established in sections not well adapted to a large patronage of patients suitable for clinical instruction, and the paucity of these forced the teachers to other methods, and an increase of time to permit their full development.

The question that interests the writer of this is not how much may be added to the curriculum, but how thoroughly that which is already engrafted upon our best colleges is taught. It is an axiom in education, as well as in other things, to "make haste slowly," and time must be given to test all methods before resorting to others the value of which remains an undetermined quantity. The effort to make dentists practically equal to doctors of medicine has led to overcrowding the curricula of all the schools to an extent that the most earnest teachers are seriously asking the question, "How is it possible that all this can be accomplished in three years?" The answer simply is, It cannot be. When due consideration is given the subjects usually taught in the best-arranged dental colleges, the mind shrinks appalled at the outlook, and no intelligent educator dares to expect that the student can, or will, be able to take more than a superficial view of the whole as a basis for his future work in the greater world of which he is to form a part. It may be said that this is true of all education. This is admitted, but it is certainly essential that the untrained student should absorb to a large degree the practical as well as the collateral theoretical branches of his profession. To accomplish this, time must be given for thorough digestion of basic principles of the entire curriculum.

This necessity in a student's experience leads up to the thought, Why spend so much time in the non-essentials sought to be inculcated in the articles and discussions laid before us by this association? To the mind of the writer, some of these are too elementary to deserve a place in the proceedings of a body of educators such as this.

Young men enter college to acquire there a profession which is peculiar, in that it combines, as no other, a certain amount of mechanical ability with a knowledge of the human organism but little less than that demanded of medical graduates. The first effort, therefore, must be to discover if that which is primarily important exists latent in the mind of the student, a talent for mechanics, the foundation work of dentistry. If this be not present in the individual, time is more than wasted in the endeavor to make him or her a dentist. In order to discover this, the student should be placed upon a work that will fully demonstrate it, and there is no better method than to start him upon metal plates in the laboratory. The manipulation of rubber will not meet this want, and yet it is upon this that colleges generally place the student of the first year. That this will not give any clear idea of the trend of mind must be apparent to the most superficial observer. In connection with this the freshmen year is largely occupied in acquiring knowledge of form by drawings, mouldings, and in printing silhouette impressions of canals. Now, it may be asked in all seriousness, Is this essential? Form may be recognized by one having a talent for drawing from the model, but to one who has it not it is, in the writer's opinion, a useless expenditure of valuable hours, while, at the same time, it fails as an indicator of the student's possible ability as a mechanician. The knowledge of form gradually grows upon the mind by daily observation of natural teeth in the course of clinical practice, and that with a positiveness that time cannot efface. The filing of teeth to secure a correct idea of the form of pulp-canals is necessary and should be obligatory, but after that is done, of what use to the student will be the silhouette pictures of the same canals? About as valuable as it would be for the student, over the cadaver, to make a drawing of every muscle dissected.

A certain amount of this preliminary work is necessary, but it must be brought, in every case, as near to the future requirements of the student as possible. It is therefore thought to be a mistake to give students the work of forming cavities in bone, and carving wood, moulding in clay, or carving soap to represent teeth. This method of instruction recalls the case of a student, familar to the writer, who began the study of dentistry with a German practitioner. He placed the young aspirant for a dental education upon carving teeth out of ivory, and kept him at this work for six months When asked, "Do you make teeth of ivory now?" he replied, "No, but you must acquire manipulative skill, and this is the best way to accomplish it."

The statement is made that the dental colleges that adopted a graded technical course were very few prior to 1893, and all those mentioned were in the West. This is an error and should be corrected as a matter of history. The school with which the writer is most familiar had for many years prior to this a graded course in technical instruction, and this continues to the present day, omitting non-essentials. When this was established it was supposed to be a part of good teaching and not worthy of special notice, and not at variance to that followed by all well-regulated schools. It comes, therefore, in the nature of a surprise to find that so many colleges were deficient in this personal preliminary supervision work. If this technic association has accomplished nothing more than inducing the schools to better methods, it will have justified its existence for the time being.

In the Eastern schools and, possibly, in all schools situated in large centres of population the demand for dental service is largely in excess of the ability of the students to perform, requiring frequently engagements several weeks in advance. The fact is recognized, it is presumed, that the so-called dummy practice stands in no relation, as regards value, to the practice on the living body. This being so, it is vitally important that, after fundamental principles are engrafted upon the mind, students should be given charge of patients with as little delay as possible. It is a grave mistake, in the writer's judgment, to practically omit this until the third year of pupilage. It is no marvel that some men go out of their respective colleges not as well prepared as they should be to assume the grave responsibilities they are sure to meet. If colleges cannot secure the living subjects for mechanical, operative, and pathological practice for students, then as dental educational institutions they have no right to exist, for the clinic is the vital point in this character of instruction, and without it dentistry cannot be taught.

There are many collateral subjects to which the student's attention should be called, but until more time is given to training than we now have—three years—these must be relegated to the future. What we most need is a higher, but not too high, entrance standard, and then thorough work in every branch allotted to our care. This requires large means, ample building room, perfect appliances, and, above all, *teachers* in the highest and best sense, and this, it is presumed, will be the most difficult problem to surmount in the future, as it is in the present.

